# Markers Indicating Body Vitamin D Stores and Responses of Liver and Adipose Tissues to Changes in Vitamin D Intake in Male Mice

**DOI:** 10.3390/nu12051391

**Published:** 2020-05-13

**Authors:** Mikis Kiourtzidis, Julia Kühn, Corinna Brandsch, Anja-Christina Baur, Monika Wensch-Dorendorf, Gabriele I. Stangl

**Affiliations:** 1 Institute of Agricultural and Nutritional Sciences, Martin Luther University Halle-Wittenberg, Von-Danckelmann-Platz 2, 06120 Halle (Saale), Germany; mikis.kiourtzidis@landw.uni-halle.de (M.K.); julia.kuehn@landw.uni-halle.de (J.K.); corinna.brandsch@landw.uni-halle.de (C.B.); anja-christina.baur@landw.uni-halle.de (A.-C.B.); monika.wensch-dorendorf@landw.uni-halle.de (M.W.-D.); 2 Competence Cluster of Cardiovascular Health and Nutrition (nutriCARD), Halle-Jena-Leipzig, Germany

**Keywords:** 25(OH)D, adipose tissue, liver, mice, vitamin D

## Abstract

Circulating 25-hydroxyvitamin D (25(OH)D) is regarded as the most reliable biomarker of vitamin D status. However, limited data exist concerning the suitability of 25(OH)D as an indicator of body vitamin D stores and the ability of adipose tissue to mobilize vitamin D. In the first study, in which male mice received different vitamin D_3_ doses for three weeks, we found strong linear response relationships between vitamin D_3_ intake and levels of vitamin D_3_ in the plasma (*p* < 0.001), liver (*p* < 0.001) and adipose tissues (*p* < 0.001), and strong positive correlations between plasma and tissue stores of vitamin D_3_ (*p* < 0.001). Plasma levels of 25(OH)D_3_ and 24,25-dihydroxyvitamin D_3_ (24,25(OH)_2_D_3_) showed weak or no correlations with tissue vitamin D_3_ stores. Data from a second study demonstrate a strong and rapid response of plasma 25(OH)D_3_ in vitamin D_3_-treated mice with a low vitamin D status. Additionally, mice fed a vitamin D-free diet showed a strong and rapid decline in vitamin D_3_ in the liver, whereas the decline in different adipose tissues was distinctly lower than that in the liver. To conclude, tissue stores of vitamin D_3_ were best reflected by plasma vitamin D_3_. In contrast to the liver, adipose tissues responded less sensitively to an absence of vitamin D intake.

## 1. Introduction

The research interest in vitamin D has increased significantly due to the high prevalence of vitamin D insufficiency worldwide [1] and the multiple functions of vitamin D besides mineral metabolism, e.g., in the immune system [2,3]. Thus, numerous epidemiological studies have been conducted linking vitamin D to health outcomes. The concentration of 25-hydroxyvitamin D (25(OH)D), which is analyzed in serum, plasma or dried blood spots by different measures, has widely been accepted as a good biomarker of vitamin D status in adults and infants [4,5]. The suitability of 25(OH)D as a vitamin D status biomarker is based on the finding that 25(OH)D has a long-term half-life of at least 2 weeks in plasma, thereby reflecting vitamin D stores in the body [6,7]. Additionally, it is assumed that the synthesis of 25(OH)D in the liver is not strictly regulated and depends on the available quantity of vitamin D [5]. In contrast to 25(OH)D, bioactive 1α,25-dihydroxyvitamin D (1,25(OH)_2_D) has been considered an inappropriate biomarker of vitamin D status because its synthesis and activity are tightly regulated by the parathyroid hormone and fibroblast growth factor 23 [8]. Both hormones ensure constant levels of plasma 1,25(OH)_2_D for a wide range of vitamin D concentrations in the body. 24,25-Dihydroxyvitamin D (24,25(OH)_2_D), which is formed from 25(OH)D and 1,25(OH)_2_D by the multicatalytic enzyme 25-hydroxyvitamin D-24-hydroxylase (CYP24A1) [9] and destined for excretion, is usually not used as a biomarker of vitamin D status. The measurement of 24,25(OH)_2_D, for example, has been recommended for the diagnosis of patients with mutations in CYP24A1 [10].

However, a few scientists recommend assessing the ratio of 25(OH)D to 24,25(OH)_2_D to evaluate vitamin D status [11,12]. Currently, few data are available regarding the suitability of nonhydroxylated vitamin D as a status marker. In the *First International Conference on Controversies in Vitamin D,* the measurement of potential vitamin D biomarkers other than 25(OH)D was discussed [13]. Recently, the reliability of 25(OH)D as a biomarker of vitamin D status has been critically discussed. In 2018, Jorde and Grimnes hypothesized that nonhydroxylated vitamin D in serum could be a more useful parameter for assessing vitamin D status [14]. The authors argued that vitamin D-dependent cells possibly prefer circulating vitamin D, not 25(OH)D or 1,25(OH)_2_D, to synthesize bioactive vitamin D metabolites for their own needs.

Additionally, data from human intervention studies indicate that the activity of the 25-hydroxylases that catalyze the synthesis of 25(OH)D from vitamin D is regulated because the increase in circulating 25(OH)D following vitamin D treatment is higher in individuals with low baseline levels of 25(OH)D than in those with high 25(OH)D levels [15,16]. Other data, which indicate that circulating levels of 25(OH)D do not necessarily reflect vitamin D stores in the body, come from mouse studies. Mice that were fed diets containing 7-dehydrocholesterol or ergosterol showed significantly increased vitamin D stores in the liver and kidney, while having unchanged serum concentrations of 25(OH)D [17,18]. Additionally, mice that received ezetimibe, an inhibitor of the sterol transporter Niemann-Pick C1-like 1 protein, had markedly reduced vitamin D stores in the liver, kidney, adipose tissues and muscle, while the circulating concentrations of 25(OH)D had increased [19].

Due to many open issues concerning the best and most sensitive biomarker reflecting tissue vitamin D stores and changes in vitamin D consumption, we conducted two mouse studies. The first study addressed response relationships between vitamin D_3_ intake, tissue levels of vitamin D_3_ and circulating concentrations of different vitamin D_3_ metabolites. The second study aimed to ascertain the response of plasma and tissue concentrations of vitamin D metabolites to changes in the oral vitamin D_3_ supply in mice with a low, adequate or high vitamin D status. Additionally, we studied the rate of mobilization of vitamin D_3_ from the liver and different types of adipose tissues in response to an absent vitamin D intake.

## 2. Materials and Methods

### 2.1. Mouse Studies

In total, two studies using male wild-type mice (C57BL/6N; Charles River, Sulzfeld, Germany) were conducted. The experimental procedures were approved by the local ethics committee (Martin Luther University Halle-Wittenberg, Germany; approval numbers: H1-4/T1-18, H1-4/T1-19; date of approval: 17 July 2018, 1 March 2019, respectively). All experimental procedures followed the guidelines for the care and handling of laboratory animals according to the US National Research Council [20]. Mice were housed in a room controlled for temperature (22 ± 2 °C), light (12-h light, 12-h dark cycle, lamps emitting no ultraviolet irradiation) and relative humidity (50–60%) and had free access to food and water. The basal diets used in these studies consisted of (per kg): 288 g starch, 200 g sucrose, 200 g casein, 175 g coconut fat, 60 g of a vitamin and mineral mixture, 50 g cellulose, 25 g soybean oil and 2 g of DL-methionine. Except for vitamin D_3_, vitamins and minerals were added to the diet according to the recommendation of the National Research Council [21].

The first experiment was conducted as a dose-response study to elucidate concentrations of vitamin D metabolites in plasma, the liver and adipose tissues (mesenteric, retroperitoneal and subcutaneous) in response to increasing doses of dietary vitamin D_3_ and to assess the most suitable plasma marker indicating tissue stores of vitamin D_3_. Therefore, 30 five-week-old mice with an initial body weight of 19.6 ± 0.6 g were randomly assigned to 10 groups (*n* = 3), receiving 5 µg, 10 µg, 15 µg, 20 µg, 25 µg, 30 µg, 35 µg, 40 µg, 45 µg or 50 µg of vitamin D_3_ per kg of diet for three weeks. The dietary vitamin D concentrations chosen for the study included levels below and above the presumed need for vitamin D.

The second study aimed to assess the extent and rate of changes in the plasma and tissue vitamin D metabolites in response to switching the vitamin D_3_ supply from low to adequate and from high to adequate. Therefore, 72 five-week-old mice (initial body weight: 18.4 ± 2.0 g) were randomly assigned to 3 groups (*n* = 24) and fed diets with either low (5 µg/kg), adequate (25 µg/kg) or high (50 µg/kg) concentrations of vitamin D_3_ for four weeks to induce different statuses of vitamin D_3_. Then, all mice received a diet containing 25 µg/kg of vitamin D_3_. Thus, the study included three intervention groups: a low vitamin D_3_ status group that received a vitamin D_3_-adequate diet (5→25 D_3_), a high vitamin D_3_ status group that received a vitamin D_3_-adequate diet (50→25 D_3_) and a control group that was fed the vitamin D_3_-adequate diet (25→25 D_3_) over the whole experimental period. Six mice from each group were analyzed for plasma and liver concentrations of vitamin D metabolites at the baseline (after the 4-week treatment with 5, 25 and 50 µg/kg vitamin D_3_, respectively) and 7, 14 and 21 days after switching the dietary vitamin D_3_ concentrations.

Additionally, to elucidate the response of circulating vitamin D metabolites and vitamin D stores in liver and adipose tissues to an absent vitamin D supply, 24 five-week-old mice (initial body weight: 17.4 ± 1.8 g), which received a vitamin D_3_-adequate diet (25 µg/kg) for 3 weeks, were placed on a vitamin D-free diet (0 µg/kg). Over a two-day interval, three mice each were analyzed for vitamin D metabolites in the plasma, liver, and mesenteric, retroperitoneal and subcutaneous adipose tissues over a period of 14 days.

### 2.2. Blood and Tissue Sampling

Prior to sampling, each mouse was food deprived for 4 h, anesthetized with diethyl ether, decapitated and exsanguinated. Blood was used for the analyses of vitamin D metabolites in whole blood, the erythrocytes and the plasma. The liver and mesenteric, retroperitoneal and subcutaneous adipose tissues were harvested, immediately snap-frozen in liquid nitrogen and subsequently stored at −80 °C until the analysis of the vitamin D metabolites.

To quantify vitamin D metabolites in the erythrocytes, blood was collected in heparinized tubes (Sarstedt, Nümbrecht, Germany) and centrifuged at 2000 × *g* for 10 min at 20 °C. The obtained plasma was removed and stored for further analyses. The erythrocyte fraction was washed three times with ice-cold isotonic sodium chloride solution and then centrifuged (2000× *g*, 10 min, 20 °C). To ensure that the erythrocytes contained no adherent vitamin D, the supernatant obtained after each washing step was analyzed for vitamin D metabolites. After the second washing cycle, all vitamin D metabolites in the supernatant were below the limit of quantitation (LOQ, vitamin D_3_: 0.1 nmol/L, 25(OH)D_3_: 0.8 nmol/L, 24,25(OH)_2_D_3_: 5.1 nmol/L).

### 2.3. Analysis of Vitamin D Metabolites

Vitamin D_3_, 25(OH)D_3_ and 24,25(OH)_2_D_3_ were quantified by liquid chromatography-tandem mass spectrometry (LC-MS/MS). Blood and tissue samples were prepared as previously described [18,19]. A mixture of 7-fold deuterated vitamin D_3_ (Toronto Research Chemicals Inc., Toronto, ON, Canada) and 6-fold deuterated 25(OH)D_3_ (Chemaphor Chemical Services, Ottawa, ON, Canada) was used as an internal standard. All samples were subjected to HPLC (1260 Infinity Series, Agilent Technologies, Waldbronn, Germany) coupled to a tandem mass spectrometer (QTRAP 5500, SCIEX, Darmstadt, Germany). The HPLC conditions have been described elsewhere [19]. For quantification of vitamin D_3_ and 25(OH)D_3_, a Hypersil ODS C18 column (120 A, 5 μm, 150 × 2.0 mm^2^, VDS Optilab, Berlin, Germany) was used, and for quantification of 24,25(OH)_2_D_3,_ a Poroshell C18 column (120 A, 2.7 μm, 50 × 4.6 mm^2^, Agilent Technologies) was used.

Ionization was achieved by positive electrospray, and data were recorded in multiple reaction monitoring mode with the following transitions: vitamin D_3_ 560 > 298, 7-fold deuterated vitamin D_3_ 567 > 298, 25(OH)D_3_ 576 > 298, 24,25(OH)_2_D_3_ 592 > 298, and 6-fold deuterated 25(OH)D_3_ 582 > 298. Metabolites were present as adducts of 4-phenyl-1,2,4-triazoline-3,5-dione (Sigma-Aldrich, Steinheim, Germany).

The intraday precisions of the analytical method for vitamin D_3_, 25(OH)D_3_ and 24,25(OH)_2_D_3_ were 5.15%, 2.07% and 12.9%, respectively, determined in pooled plasma samples.

The circulating concentration of 1,25(OH)_2_D was analyzed by a commercial enzyme-linked immunoassay (Immunodiagnostic Systems, Frankfurt am Main, Germany) following the procedure given by the manufacturer with modifications [17].

### 2.4. Statistical Analysis

Statistical analysis was performed using the SAS 9.4 software package (SAS Institute Inc., Cary, NC, USA). Least-squares means (LSM) were estimated using the MIXED procedure, and the differences in LSM were tested for significance. LSM were considered to be significantly different at *p* < 0.05. Regression models (M1, M1a) were fitted using PROC REG and PROC NLIN. Depending on the experimental design and data structure, one of the following models was used for the measured traits:

*y_ij_* = *a* + *b*·*x_ij_* + *e_ij_* (M1)

*y_ij_* = *a* + *b*·*x_ij_* + *c*·(*x_ij_*·*x_ij_*) + *e_ij_*, for *x_ij_* < *x*_0_ and *y_ij_* = *plateau* + *e_ij_*, for *x_ij_* ≥ *x*_0_ (M1a)

*y_ijk_* = *μ* + *diet_i_* + *time_j_* + (*diet* × *time*)*_ij_* + *e_ijk_* (M2)

*y_ij_* = *μ* + *time_i_* + *e_ij_* (M3)

Regression models (M1, M1a) were used to model the traits of the dose-response study (Section 2.1) depending on vitamin D_3_ doses ranging from 5 to 50 µg/kg of diet with 5 µg/kg increments and 3 mice per dose. The M1 model used a linear regression, and M1a used a curvilinear-plateau model [22]. Model M2 was used to analyze the diet and time effects, including the 2-way interactions for the second study described in Section 2.1. Model M3 was used to analyze the time effect for the last study described in Section 2.1.

The factors diet and time as well as their interaction were considered fixed effects in the models. The F-test was used to assess differences in fixed effects levels *(p* < 0.05). Finally, the Tukey adjusted *t*-test (for main effects with more than 2 levels) or a *t*-test (main effects with 2 levels and interactions) was used to assess pairwise differences (*p* < 0.05). To assess correlations between vitamin D metabolites in plasma and stores of vitamin D in tissues, Pearson correlation coefficients were calculated using the CORR procedure.

## 3. Results

### 3.1. First Mouse Study

**Figure 1:**
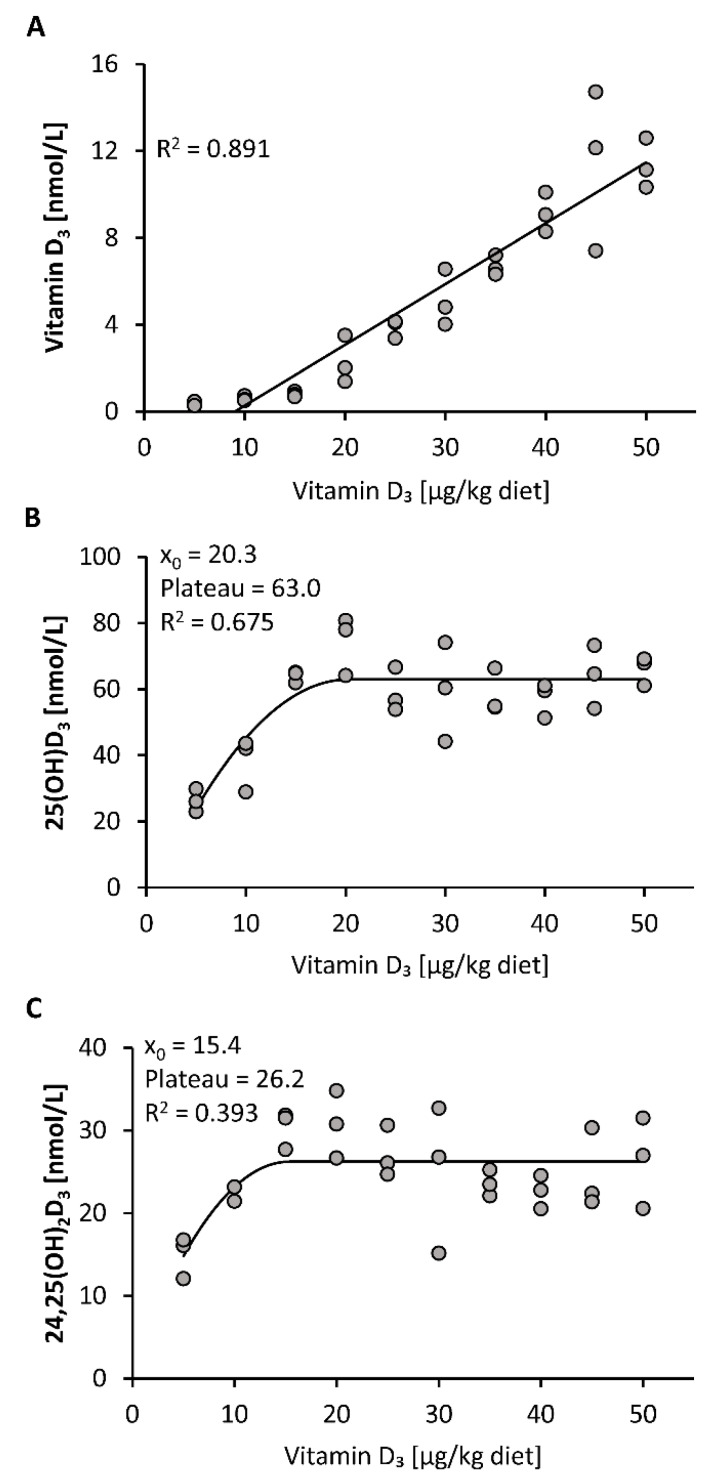
Plasma concentrations of vitamin D_3_ (**A**), 25(OH)D_3_ (**B**) and 24,25(OH)_2_D_3_ (**C**) in mice that were fed 10 different doses of dietary vitamin D_3_ for three weeks (*n* = 3). The regression line is linear for vitamin D_3_ (**A**) and curvilinear-plateau for 25(OH)D_3_ (**B**) and 24,25(OH)_2_D_3_ (**C**). 25(OH)D_3_, 25-hydroxyvitamin D_3_; 24,25(OH)_2_D_3_, 24,25-dihydroxyvitamin D_3_; R^2^, squared correlation; x_0_, the x value at which the curve merges into the plateau.

All mice included in this study gained body weight during the 3-week treatment without showing any differences in the final body weights between the 10 groups of mice (mean body weight 23.6 ± 0.9 g, *n* = 30). First, we investigated the response of plasma vitamin D_3_ metabolites to increasing doses of dietary vitamin D_3_. The data show a linear increase in the plasma concentration of vitamin D_3_ in response to feeding increasing doses of vitamin D_3_, ranging from 5 to 50 µg/kg (Figure 1A). In contrast, plasma concentrations of 25(OH)D_3_ and 24,25(OH)_2_D_3_ showed a disproportionally strong increase in the low-dose ranges of dietary vitamin D_3_ and a plateau-like response near the presumed need for vitamin D_3_ (Figure 1B,C).

**Figure 2:**
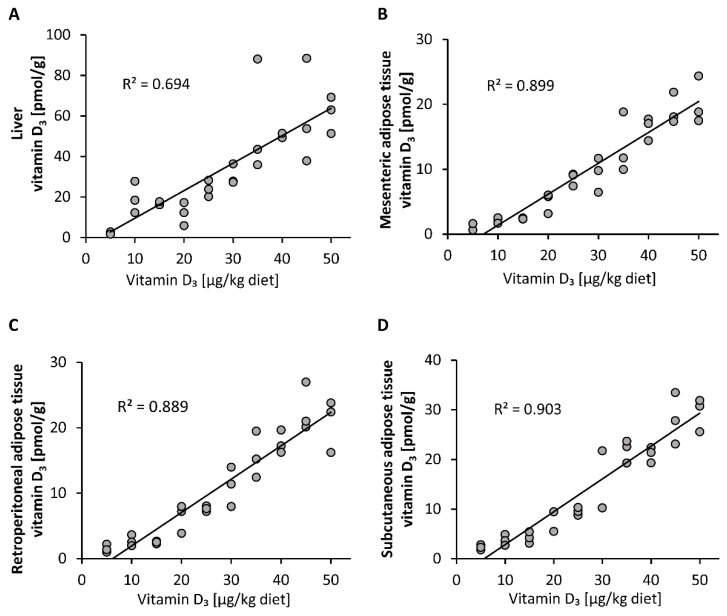
Concentrations of vitamin D_3_ in the liver (**A**) and mesenteric (**B**), retroperitoneal (**C**) and subcutaneous (**D**) adipose tissues of mice that were fed 10 different doses of dietary vitamin D_3_ for three weeks (*n* = 3). The regression line is linear for vitamin D_3_ in the analyzed tissues. R^2^, squared correlation.

Compared to the plasma vitamin D_3_ responses, the vitamin D_3_ stores in liver, mesenteric, retroperitoneal and subcutaneous adipose tissues showed a linear dose-response to increasing doses of vitamin D_3_ (Figure 2A–D). The concentrations of 25(OH)D_3_ and 24,25(OH)_2_D_3_ in all groups and tissues analyzed were below the LOQ (25(OH)D_3_: liver: 2.5 pmol/g, mesenteric adipose tissue: 2.8 pmol/g, retroperitoneal adipose tissue: 3.3 pmol/g, subcutaneous adipose tissue: 2.8 pmol/g; 24,25(OH)_2_D_3_: liver: 5.0 pmol/g, mesenteric adipose tissue: 7.6 pmol/g, retroperitoneal adipose tissue: 8.9 pmol/g, subcutaneous adipose tissue: 7.4 pmol/g).

**Figure 3:**
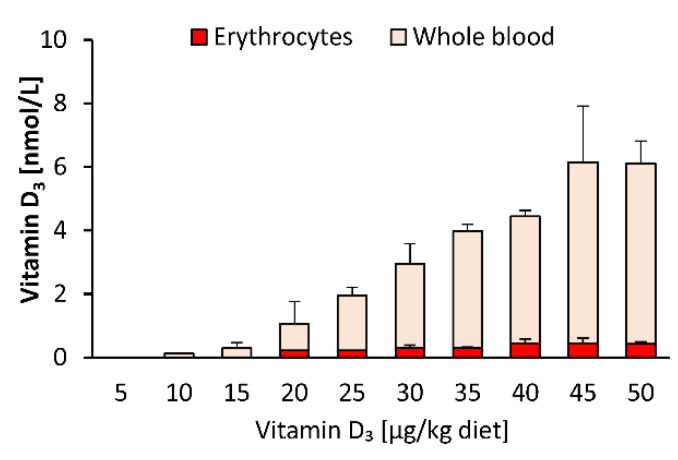
Concentrations of vitamin D_3_ in erythrocytes and whole blood of mice that were fed 10 different doses of dietary vitamin D_3_ for three weeks (*n* = 3). Data are presented as the means ± SD.

As blood spot samples are also used for vitamin D status analysis, we compared the response of vitamin D_3_ in whole blood and isolated blood cells to feeding different doses of dietary vitamin D_3_. Figure 3 illustrates that the proportion of vitamin D_3_ in the blood cells (mainly erythrocytes) was distinctly lower than that in whole blood, indicating that most of the circulating vitamin D_3_ was allocated to plasma, and that erythrocytes may not serve as a vitamin D store. The concentrations of 25(OH)D_3_ and 24,25(OH)_2_D_3_ in erythrocytes were below the LOQ (25(OH)D_3_: 7.9 nmol/L; 24,25(OH)_2_D_3_: 9.6 nmol/L) in each group of mice.

**Figure 4:**
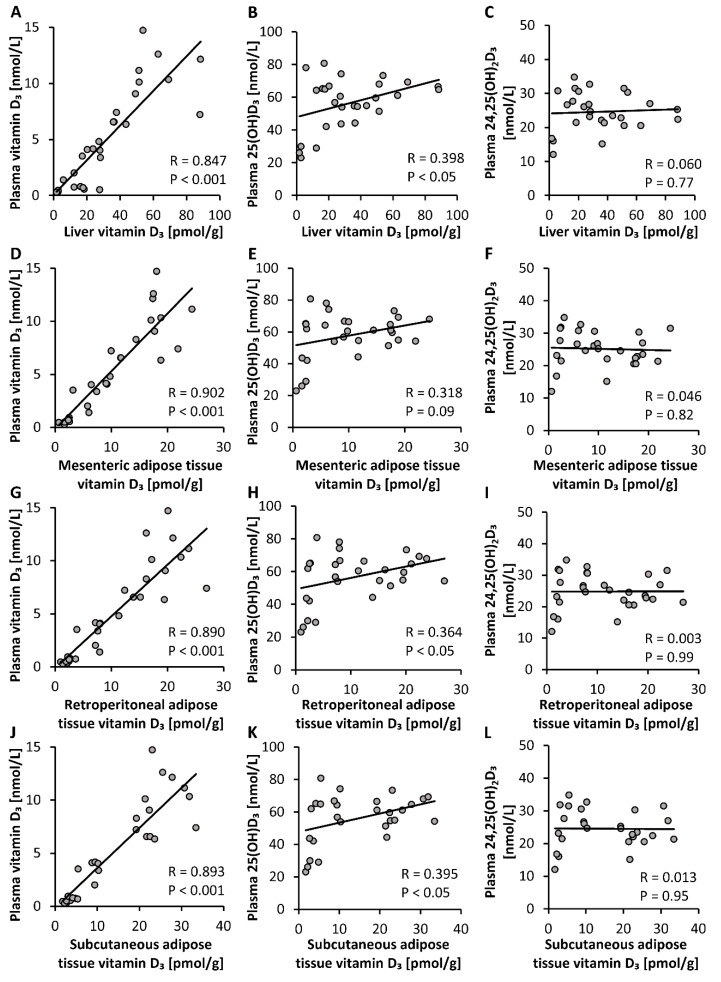
Correlations between concentrations of plasma vitamin D_3_ metabolites and vitamin D_3_ stores in the liver (**A**–**C**) and mesenteric (**D**–**F**), retroperitoneal (**G**–**I**) and subcutaneous (**J**–**L**) adipose tissues of mice that were fed diets containing 5 µg, 10 µg, 15 µg, 20 µg, 25 µg, 30 µg, 35 µg, 40 µg, 45 µg or 50 µg vitamin D_3_ per kg of diet for three weeks (*n* = 3). 25(OH)D_3_, 25-hydroxyvitamin D_3_; 24,25(OH)_2_D_3_, 24,25-dihydroxyvitamin D_3_; R, correlation coefficient.

To elucidate the most suitable plasma vitamin D metabolite that may serve as an indicator of tissue vitamin D stores, we looked for correlations between circulating levels of different vitamin D_3_ metabolites and tissue stores of vitamin D_3_. Analyses revealed that levels of plasma vitamin D_3_ showed a strong correlation with vitamin D_3_ stores in liver, mesenteric, retroperitoneal and subcutaneous adipose tissues (*p* < 0.001, Figure 4A,D,G,J). However, the plasma concentration of 25(OH)D_3_ showed only weak correlations with vitamin D_3_ stores in liver, retroperitoneal and subcutaneous adipose tissues (*p* < 0.05, Figure 4B,H,K) and no correlation with vitamin D_3_ in mesenteric adipose tissue (*p* > 0.05, Figure 4E). Plasma levels of 24,25(OH)_2_D_3_ did not correlate with vitamin D_3_ stores in any tissues analyzed (*p* > 0.05, Figure 4C,F,I,L).

### 3.2. Second Mouse Study

**Figure 5:**
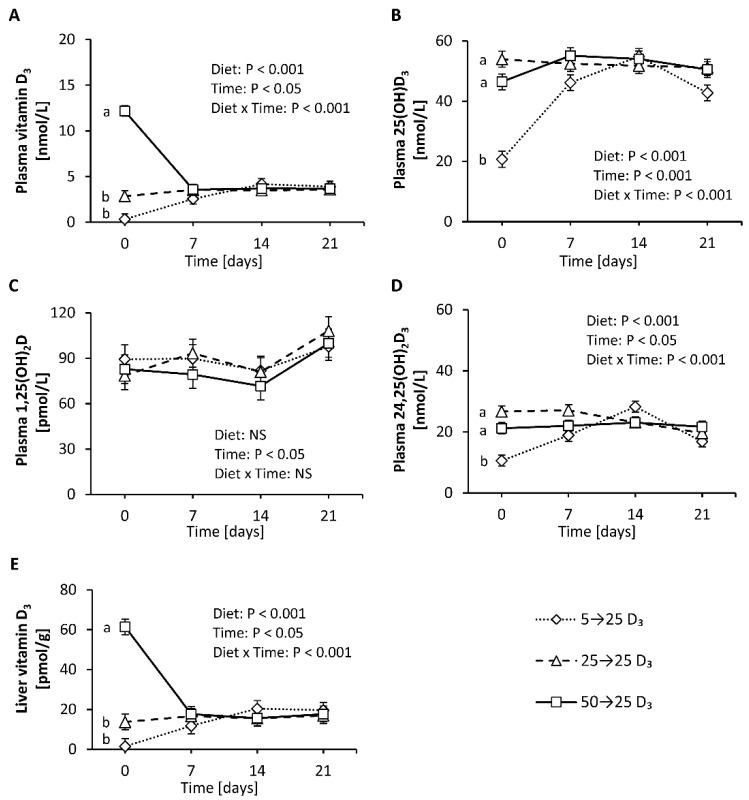
Concentrations of vitamin D_3_ (**A**), 25(OH)D_3_ (**B**), 1,25(OH)_2_D (**C**) and 24,25(OH)_2_D_3_ (**D**) in plasma and vitamin D_3_ in the liver (**E**) of mice with a low, adequate or high vitamin D status (induced by feeding diets with 5 µg/kg, 25 µg/kg, and 50 µg/kg vitamin D_3_, respectively, for four weeks) that received diets with 25 µg/kg vitamin D_3_ for three weeks. Six mice per group were used for the analysis of plasma and liver vitamin D metabolites at baseline (day 0) and 7, 14 and 21 days each. Data are presented as least-squares means (LSM) ± standard error of LSM (*n* = 6). Different letters indicate significant differences between the groups at a given time point. 5→25 D_3_, low vitamin D_3_ status group that received a vitamin D_3_-adequate diet; 50→25 D_3_, high vitamin D_3_ status group that received a vitamin D_3_-adequate diet; 25→25 D_3_, control group that was fed a vitamin D_3_-adequate diet over the whole experimental period; 25(OH)D_3_, 25-hydroxyvitamin D_3_; 1,25(OH)_2_D, 1α,25-dihydroxyvitamin D; 24,25(OH)_2_D_3_, 24,25-dihydroxyvitamin D_3_; NS, not significant.

First, this study aimed to elucidate the response of vitamin D metabolites in plasma to adequate amounts of dietary vitamin D_3_ in mice having a low, adequate or high vitamin D status that was induced by a 4-week treatment with diets containing 5 µg/kg (low), 25 µg/kg (adequate) and 50 µg/kg (high) of vitamin D_3_. The final body weights of the mice were not affected by the different treatments (group 5→25 D_3_: 28.6 ± 2.3 g, group 25→25 D_3_: 30.3 ± 1.7 g, group 50→25 D_3_: 27.0 ± 2.1 g). Figure 5 illustrates that the plasma concentrations of 25(OH)D_3_ and 24,25(OH)_2_D_3_ markedly and rapidly increased in mice with a low vitamin D status that were treated with an adequate dose of vitamin D_3_ (Figure 5B,D), while the concentration of vitamin D_3_ in the plasma and liver showed a moderate increase (Figure 5A,E). In contrast, the level of vitamin D_3_ in the plasma and liver declined rapidly and strongly when mice with a high vitamin D status were fed an adequate dose of vitamin D_3_ (Figure 5A,E), whereas the plasma concentration of 25(OH)D_3_ slightly increased (Figure 5B) and that of 24,25(OH)_2_D_3_ remained unchanged (Figure 5D). The concentration of plasma 1,25(OH)_2_D did not show significant differences between the treatments (Figure 5C). The vitamin D metabolites in the plasma and liver of control mice with an adequate vitamin D status that received an adequate dose of vitamin D_3_ over the experimental period remained largely unaltered (Figure 5A–E).

**Figure 6:**
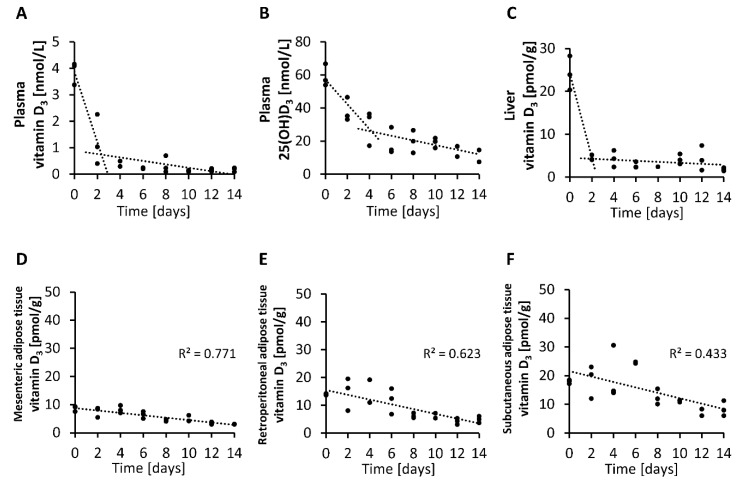
Concentrations of vitamin D_3_ (**A**) and 25(OH)D_3_ (**B**) in plasma and vitamin D_3_ in the liver (**C**) and mesenteric (**D**), retroperitoneal (**E**) and subcutaneous (**F**) adipose tissues measured over two-day intervals from mice that were placed on a vitamin D-free diet (0 µg/kg). Day 0 represents the baseline levels of mice that were fed 25 µg/kg vitamin D_3_ with their diet (*n* = 3). 25(OH)D_3_, 25-hydroxyvitamin D_3_; R^2^, squared correlation.

Finally, we analyzed the rate of decline in circulating and tissue levels of vitamin D_3_ over two-day intervals in mice with an adequate vitamin D status that were placed on a vitamin D-free diet. The findings demonstrate that the level of vitamin D_3_ in the plasma and liver declined strongly and rapidly within 2 to 4 days after feeding the vitamin D-free diet (Figure 6A,C), while the plasma concentration of 25(OH)D_3_ showed a more moderate decline after subjecting mice to a vitamin D-free diet (Figure 6B). In contrast, when looking at the vitamin D_3_ levels in the adipose tissues, we found small and more linear reductions over time in the mesenteric, retroperitoneal and subcutaneous adipose tissues (Figure 6D–F).

## 4. Discussion

It is a consensus that circulating 25(OH)D is the most representative measure for vitamin D status [23,24,25]. 25(OH)D is primarily used for the diagnosis of vitamin D deficiency and to evaluate associations between vitamin D status and disease morbidity and mortality in epidemiological studies. The question, however, arose whether circulating 25(OH)D may also reflect the quantity of stored vitamin D. This is important with respect to the fact that stored vitamin D, particularly in the liver and adipose tissue, has been suggested to serve as an important source of vitamin D that can be mobilized in times of absent endogenous vitamin D synthesis or low vitamin D intake to counteract vitamin D insufficiency [26,27]. The contribution of stored vitamin D to improving vitamin D status became evident in a prospective, double-blind cohort study that was published in 2017 [28]. In this human study, large subcutaneous adipose tissue stores of vitamin D were associated with a reduced decline in serum 25(OH)D concentrations in the following year. This finding suggests the importance of stored vitamin D in reducing the risk of vitamin D insufficiency. Having a good serum biomarker that reliably indicates vitamin D stores would, therefore, be important for public health and clinical practice. As it is difficult to assess the liver and adipose tissue stores of vitamin D in humans, we conducted two studies in mice.

The important findings of the first study were that the plasma, liver and adipose tissue concentrations of vitamin D_3_ increased linearly with increasing doses of orally administered vitamin D_3_, and that the circulating vitamin D_3_ strongly correlated with the vitamin D_3_ stores in the liver and all adipose tissues analyzed (R between 0.85 and 0.90). However, current data also show that blood cells, in particular erythrocytes, may not serve as important vitamin D stores. In contrast to vitamin D_3_, circulating 25(OH)D_3_ showed only a weak correlation (R between 0.36 and 0.40), and 24,25(OH)_2_D_3_ showed no correlation with vitamin D_3_ in the liver and adipose tissues. Therefore, it seems that neither vitamin D metabolite is an ideal parameter reflecting tissue stores of vitamin D in the body.

The weak correlation between circulating 25(OH)D_3_ and the tissue stores of vitamin D_3_ is attributable to the fact that the response of 25(OH)D_3_ to increasing doses of vitamin D_3_ does not follow a linear relationship. Current data from a dose-relationship study in mice and several findings from human studies (e.g., [29,30]) show a disproportional increase in circulating 25(OH)D following increasing vitamin D doses. In general, dose-response curves of nutrient status markers normally follow a quadratic or curvilinear function and reach a transient plateau, or flattening of the curve, when the nutrient need is covered. Thus, the observed curvilinear-plateau response of 25(OH)D_3_ to increasing vitamin D_3_ doses indicates regulation and makes 25(OH)D a sensitive biomarker of low and adequate vitamin D status. Gallagher et al. assume that the curvilinear plateau of 25(OH)D is a result of an increased degradation of 25(OH)D to 24,25(OH)_2_D [31]. However, as we also observed a plateau in 24,25(OH)_2_D_3_ despite increasing vitamin D_3_ doses, we assume that 25(OH)D is not primarily regulated via its degradation to 24,25(OH)_2_D but rather by hepatic synthesis. This contradicts, at least in part, the common view that the production of 25(OH)D is not significantly regulated and primarily depends on the availability of vitamin D [5]. Another interesting finding of the current study was the observation that 25(OH)D_3_ showed no decline when switching mice from a vitamin D_3_-rich diet to a vitamin D_3_-adequate diet. Based on the findings that circulating levels of 25(OH)D do not linearly respond to vitamin D intake and do not necessarily reflect tissue stores of vitamin D, the significance and reliability of analyzed plasma 25(OH)D for classifying vitamin D deficiency/inadequacy is limited. The measurement of plasma vitamin D could provide additional and more reliable information concerning vitamin D uptake and the body’s stores of vitamin D than 25(OH)D. Thus, plasma vitamin D could serve as a clinically relevant parameter to estimate the body’s stores of vitamin D in humans. The clinical implementation of plasma vitamin D analysis could be used to assess depleted vitamin D stores or to avoid an unnecessary use of vitamin D supplements. However, it must be noted that data on human tissue stores of vitamin D and the ability of mobilization are currently insufficient. Further studies are necessary to elucidate cut-off values of vitamin D that indicate adequate or insufficient tissue storages of vitamin D.

To elucidate the suitability of liver and adipose tissues to mobilize vitamin D in times of an absent vitamin D supply, we investigated the decline in vitamin D_3_ in the tissues of mice consuming no vitamin D. Here, we found a rapid and strong reduction in vitamin D_3_ in the liver that corresponded to the decline in vitamin D_3_ in plasma. However, in contrast to plasma vitamin D_3_, 25(OH)D_3_ showed a less rapid decline to a vitamin D-free diet, which is suggested to be a result of stimulated hepatic synthesis of 25(OH)D and possibly by the longer half-life of 25(OH)D compared to vitamin D [6,7]. In contrast to that in the liver, vitamin D_3_ in adipose tissues showed a weak and continuous reduction after the consumption of a vitamin D-free diet. The data suggest that adipose tissues can principally release vitamin D, but in contrast to the liver, this mobilization is lower and less rapid. This is an interesting finding because the vitamin D_3_ levels in the adipose tissues increased to a similar extent as the vitamin D_3_ levels in the liver in response to the feeding of increasing doses of vitamin D_3_.

The observation that adipose tissues may contribute to maintaining circulating vitamin D and 25(OH)D to only a minor extent was also shown in a study conducted with obese patients who underwent a gastric bypass [32]. The authors of this study concluded that vitamin D in adipose tissue does not significantly contribute to circulating 25(OH)D, although the individuals included in that study showed a marked loss of body fat [32].

The role of adipose tissues in vitamin D metabolism is a fundamental question because of the high prevalence of overweight and obesity worldwide [33]. In the first study, we observed a linear increase in vitamin D_3_ in the liver and adipose tissues with increasing doses of dietary vitamin D_3_. Interestingly, the storage of vitamin D_3_ in adipose tissues has already been started when feeding low doses of vitamin D_3_ that are noticeably below the requirement. If vitamin D is trapped in adipose tissue and not released, if necessary, the question will arise as to why adipose tissue has this great potential to store vitamin D, although it appears not to take part significantly in combating vitamin D deficiency. However, the current data assume that vitamin D in adipose tissues is not completely trapped and can be released, although to a significantly lesser extent and much more slowly than vitamin D in the liver. It is possible that the decline in vitamin D in adipose tissues is merely a consequence of adipose tissue turnover associated with the release of vitamin D that can be used for 25(OH)D synthesis.

To conclude, vitamin D status is currently assessed by the measurement of only one vitamin D metabolite: 25(OH)D. Here, we found that tissue stores of vitamin D_3_ were best reflected by circulating vitamin D, not by 25(OH)D. Importantly, we observed that adipose tissues can release vitamin D as their vitamin D concentrations decrease continuously in cases of an absent vitamin D consumption, but in contrast to the liver this mobilization was comparatively very small. The measurement of plasma vitamin D can be a valuable tool to estimate the body’s vitamin D store, and help to prevent a pending decline in vitamin D status or to avoid an unnecessary use of vitamin D supplements.

## Abbreviations

25(OH)D	25-hydroxyvitamin D
1,25(OH)_2_D	1α,25-dihydroxyvitamin D
24,25(OH)_2_D	24,25-dihydroxyvitamin D
CYP24A1	25-hydroxyvitamin D-24-hydroxylase
LOQ	limit of quantitation
LSM	least-squares means

## References

[1] Roth D.E., Abrams S.A., Aloia J., Bergeron G., Bourassa M.W., Brown K.H., Calvo M.S., Cashman K.D., Combs G., De-Regil L.M. (2018). Global prevalence and disease burden of vitamin D deficiency: A roadmap for action in low- and middle-income countries. Ann. N. Y. Acad. Sci..

[2] Gil Á., Plaza-Diaz J., Mesa M.D. (2018). Vitamin D: Classic and novel actions. Ann. Nutr. Metab..

[3] Umar M., Sastry K.S., Chouchane A.I. (2018). Role of vitamin D beyond the skeletal function: A review of the molecular and clinical studies. Int. J. Mol. Sci..

[4] Cashman K.D., van den Heuvel E.G., Schoemaker R.J., Prévéraud D.P., Macdonald H.M., Arcot J. (2017). 25-hydroxyvitamin D as a biomarker of vitamin D status and its modeling to inform strategies for prevention of vitamin D deficiency within the population. Adv. Nutr..

[5] Zerwekh J.E. (2008). Blood biomarkers of vitamin D status. Am. J. Clin. Nutr..

[6] Jones K.S., Assar S., Harnpanich D., Bouillon R., Lambrechts D., Prentice A., Schoenmakers I. (2014). 25(OH)D_2_ half-life is shorter than 25(OH)D_3_ half-life and is influenced by DBP concentration and genotype. J. Clin. Endocrinol. Metab..

[7] Batchelor A.J., Compston J.E. (1983). Reduced plasma half-life of radio-labelled 25-hydroxyvitamin D_3_ in subjects receiving a high-fibre diet. Br. J. Nutr..

[8] Christakos S., Dhawan P., Verstuyf A., Verlinden L., Carmeliet G. (2016). Vitamin D: Metabolism, molecular mechanism of action, and pleiotropic effects. Physiol. Rev..

[9] Beckman M.J., Tadikonda P., Werner E., Prahl J., Yamada S., DeLuca H.F. (1996). Human 25-hydroxyvitamin D_3_-24-hydroxylase, a multicatalytic enzyme. Biochemistry.

[10] Tang J.C.Y., Nicholls H., Piec I., Washbourne C.J., Dutton J.J., Jackson S., Greeves J., Fraser W.D. (2017). Reference intervals for serum 24,25-dihydroxyvitamin D and the ratio with 25-hydroxyvitamin D established using a newly developed LC-MS/MS method. J. Nutr. Biochem..

[11] Cashman K.D., Hayes A., Galvin K., Merkel J., Jones G., Kaufmann M., Hoofnagle A.N., Carter G.D., Durazo-Arvizu R.A., Sempos C.T. (2015). Significance of serum 24,25-dihydroxyvitamin D in the assessment of vitamin D status: A double-edged sword?. Clin. Chem..

[12] Kaufmann M., Gallagher J.C., Peacock M., Schlingmann K.-P., Konrad M., Deluca H.F., Sigueiro R., Lopez B., Mourino A., Maestro M. (2014). Clinical utility of simultaneous quantitation of 25-hydroxyvitamin D and 24,25-dihydroxyvitamin D by LC-MS/MS involving derivatization with DMEQ-TAD. J. Clin. Endocrinol. Metab..

[13] Sempos C.T., Heijboer A.C., Bikle D.D., Bollerslev J., Bouillon R., Brannon P.M., Deluca H.F., Jones G., Munns C.F., Bilezikian J.P. (2018). Vitamin D assays and the definition of hypovitaminosis D: Results from the First International Conference on Controversies in Vitamin D. Br. J. Clin. Pharmacol..

[14] Jorde R., Grimnes G. (2018). Serum cholecalciferol may be a better marker of vitamin D status than 25-hydroxyvitamin D. Med. Hypotheses.

[15] Waterhouse M., Tran B., Armstrong B.K., Baxter C., Ebeling P.R., English D.R., Gebski V., Hill C., Kimlin M.G., Lucas R.M. (2014). Environmental, personal, and genetic determinants of response to vitamin D supplementation in older adults. J. Clin. Endocrinol. Metab..

[16] Didriksen A., Grimnes G., Hutchinson M.S., Kjærgaard M., Svartberg J., Joakimsen R.M., Jorde R. (2013). The serum 25-hydroxyvitamin D response to vitamin D supplementation is related to genetic factors, BMI, and baseline levels. Eur. J. Endocrinol..

[17] Baur A.C., Kühn J., Brandsch C., Hirche F., Stangl G.I. (2019). Intake of ergosterol increases the vitamin D concentrations in serum and liver of mice. J. Steroid Biochem. Mol. Biol..

[18] Kühn J., Hirche F., Geissler S., Stangl G.I. (2016). Oral intake of 7-dehydrocholesterol increases vitamin D_3_ concentrations in the liver and kidney. J. Steroid Biochem. Mol. Biol..

[19] Kiourtzidis M., Kühn J., Schutkowski A., Baur A.C., Hirche F., Stangl G.I. (2020). Inhibition of Niemann-Pick C1-like protein 1 by ezetimibe reduces uptake of deuterium-labeled vitamin D in mice. J. Steroid Biochem. Mol. Biol..

[20] National Research Council (2011). Guide for the Care and Use of Laboratory Animals.

[21] National Research Council (1995). Nutrient Requirements of Laboratory Animals.

[22] Robbins K.R., Saxton A.M., Southern L.L. (2006). Estimation of nutrient requirements using broken-line regression analysis. J. Anim. Sci..

[23] European Food Safety Authority Panel on Dietetic Products, Nutrition and Allergies (2016). Scientific opinion on dietary reference values for vitamin D. EFSA J..

[24] German Nutrition Society (2012). New reference values for vitamin D. Ann. Nutr. Metab..

[25] Ross A.C., Manson J.E., Abrams S.A., Aloia J.F., Brannon P.M., Clinton S.K., Durazo-Arvizu R.A., Gallagher J.C., Gallo R.L., Jones G. (2011). The 2011 report on dietary reference intakes for calcium and vitamin D from the Institute of Medicine: What clinicians need to know. J. Clin. Endocrinol. Metab..

[26] Holick M.F. (2007). Vitamin D deficiency. N. Engl. J. Med..

[27] Heaney R.P., Davies K.M., Chen T.C., Holick M.F., Barger-Lux M.J. (2003). Human serum 25-hydroxycholecalciferol response to extended oral dosing with cholecalciferol. Am. J. Clin. Nutr..

[28] Martinaityte I., Kamycheva E., Didriksen A., Jakobsen J., Jorde R. (2017). Vitamin D stored in fat tissue during a 5-year intervention affects serum 25-hydroxyvitamin D levels the following year. J. Clin. Endocrinol. Metab..

[29] Ekwaru J.P., Zwicker J.D., Holick M.F., Giovannucci E., Veugelers P.J. (2014). The importance of body weight for the dose response relationship of oral vitamin D supplementation and serum 25-hydroxyvitamin D in healthy volunteers. PLoS ONE.

[30] Cashman K.D., Hill T.R., Lucey A.J., Taylor N., Seamans K.M., Muldowney S., Fitzgerald A.P., Flynn A., Barnes M.S., Horigan G. (2008). Estimation of the dietary requirement for vitamin D in healthy adults. Am. J. Clin. Nutr..

[31] Gallagher J.C., Sai A., Templin T., Smith L. (2012). Dose response to vitamin D supplementation in postmenopausal women: A randomized trial. Ann. Intern. Med..

[32] Pramyothin P., Biancuzzo R.M., Lu Z., Hess D.T., Apovian C.M., Holick M.F. (2011). Vitamin D in adipose tissue and serum 25-hydroxyvitamin D after roux-en-Y gastric bypass. Obesity.

[33] NCD Risk Factor Collaboration (2017). Worldwide trends in body-mass index, underweight, overweight, and obesity from 1975 to 2016: A pooled analysis of 2416 population-based measurement studies in 128·9 million children, adolescents, and adults. Lancet.

